# Netrin-1 Ameliorates Blood-Brain Barrier Impairment Secondary to Ischemic Stroke via the Activation of PI3K Pathway

**DOI:** 10.3389/fnins.2017.00700

**Published:** 2017-12-12

**Authors:** Jian Yu, Chenguang Li, Qiao Ding, Jiali Que, Kejia Liu, Haoyue Wang, Songjie Liao

**Affiliations:** Department of Neurology, Guangdong Key Laboratory for Diagnosis and Treatment of Major Neurological Diseases, National Key Clinical Department, National Key Discipline, the First Affiliated Hospital of Sun Yat-Sen University, Guangzhou, China

**Keywords:** blood-brain barrier, ischemia, netrin-1, phosphatidylinositol 3 kinase, autophagy, thalamus

## Abstract

Secondary impairment of blood-brain barrier (BBB) occurs in the remote thalamus after ischemic stroke. Netrin-1, an axonal guidance molecule, presents bifunctional effects on blood vessels through receptor-dependent pathways. This study investigates whether netrin-1 protects BBB against secondary injury. Netrin-1 (600 ng/d for 7 days) was intracerebroventricularly infused 24 h after middle cerebral artery occlusion (MCAO) in hypertensive rats. Neurological function was assessed 8 and 14 days after MCAO, and the permeability of BBB in the ipsilateral thalamus was detected. The viability of brain microvascular endothelial cells was determined after being disposed with netrin-1 (50 ng/mL) before oxygen-glucose deprivation (OGD). The role of netrin-1 was further explored by examining its receptors and their function. We found that netrin-1 infusion improved neurological function, attenuated secondary impairment of BBB by up-regulating the levels of tight junction proteins and diminishing extravasation of albumin, with autophagy activation 14 days after MCAO. Netrin-1 also enhanced cell survival and autophagy activity in OGD-treated cells, inhibited by UNC5H2 siRNA transfection. Furthermore, the beneficial effects of netrin-1 were suppressed by PI3K inhibitors 3-Methyladenine and LY294002. Our results showed that netrin-1 ameliorated BBB impairment secondary to ischemic stroke by promoting tight junction function and endothelial survival. PI3K-mediated autophagy activation depending on UNC5H2 receptor could be an underlying mechanism.

## Introduction

Vascular impairment and cellular damage take place not only in primary lesion, but also in remote loci connected to the cerebral infarction by synapses, which could be responsible for poor neurological recovery after stroke or injury (Ling et al., 2009; Zhang et al., 2012; Duering et al., 2015). The thalamus is a representative remote region connected to the ipsilateral somatosensory area of frontoparietal cortex via thalamocortical and corticothalamic projections in sensing body position and maintenance of postural reflex. Our previous study in hypertension, a well-known risk factor of stroke, has revealed vascular impairment in the ipsilateral thalamus at acute stage of focal cerebral infarction (Li et al., 2011). Therefore, improvement of vascular perfusion in the thalamus may provide a potential therapy strategy to stroke.

Angiogenesis, a sprouting process of new capillaries from preexisting ones to supply cells with oxygen, nutrients or trophic factors, helps to restore blood flow and eliminate necrotic debris in ischemic zone, and promote the survival of neurons as a result (Seevinck et al., 2010; Xiong et al., 2010; Liman and Endres, 2012). However, local newly-formed blood vessels with immature blood-brain barrier (BBB), which are characterized by high permeability, could also spark off neuronal loss due to the injury of extravasation of serum macromolecules and inflammatory factors to brain parenchyma (Abraham et al., 2002; Krueger et al., 2015; Prakash and Carmichael, 2015). The underlying mechanisms need to be clarified.

Accumulated evidence indicates that the crosstalk between the nervous and vascular systems occurs at molecular level in both development and response to injury (Weinstein, 2005). In specific, netrin-1, known as a bifunctional molecule, was originally described to mediate chemoattraction and chemorepulsion of axonal guidance or migration of neurons by interacting with its receptors, deleted in colorectal cancer (DCC) and uncoordinated gene 5H (UNC5H) (Moore et al., 2007; Rajasekharan and Kennedy, 2009). Nevertheless, netrin-1 also serves as a survival factor, which presents its pro-angiogenic activity by stimulating the proliferation, migration and differentiation of endothelial cells (ECs) into new capillary tubes, and enhancing the response of ECs to vascular endothelial growth factor (VEGF) (Nguyen and Cai, 2006; Wilson et al., 2006; Navankasattusas et al., 2008). Further studies found that netrin-1 enhanced cell migration by activating phosphatidylinositol 3 kinase (PI3K) signal cascades via UNC5B receptor (Tang et al., 2008; Lv et al., 2015). However, it is unclarified whether netrin-1 can protect vascular function in remote loci against secondary injury, and whether PI3K pathway is an unrecognized mechanism underlying the role of netrin-1.

The present study was taken to observe the temporal process of BBB impairment secondary to ischemic stroke, and to investigate whether netrin-1 could protect BBB against secondary injury *in vivo* and *in vitro*. We further explored the possible involvement of PI3K pathway, which is also a critical regulator of autophagy.

## Materials and methods

### Animal models and grouping

The experimental protocol conformed to the Animal Welfare Act Guide for Use and Care of Laboratory Animals, and was approved by the local ethics committee for animal research, Sun Yat-sen University, China. All procedures involving animals were monitored in compliance with the ARRIVE guidelines. Anesthesia for all surgical procedures was achieved using an injection of 10% chloral hydrate (3 mL/kg, i.p.).

Renovascular hypertension was firstly induced by bilateral renal artery clipping in male Sprague–Dawley rats weighing 80–100 g as described previously (Zeng et al., 1998). Twelve weeks later, 96 rats with stable hypertension (≥180 mmHg) were used in the study; rats were otherwise healthy and weighed 360–480 g. Thereafter, right cerebral infarction was established in 72 rats by electrocoagulation of the distal middle cerebral artery. This permanent middle cerebral artery occlusion (MCAO) method in hypertensive rats achieved a high consistency in the location and size of cerebral infarction, and was extensively used in the study of secondary injury post stroke (Zhang et al., 2012; Liao et al., 2013; Chen et al., 2014). There were 24 rats in the sham MCAO group, in which the right middle cerebral artery was exposed without any electrocoagulation or transection. To minimize operation time, tracheal intubation was not performed and an arterial line was not created. During recovery from anesthesia, respiratory status was in a smooth condition and body temperature was maintained at 37°C with a heating pad.

Rats with permanent MCAO were randomly allocated to receive continuous intracerebroventricular infusions of 50 μg/mL netrin-1 (cat. No. 1109-N1/CF; R&D System; *n* = 24), 50 μg/mL netrin-1 plus 200 nmoL PI3K inhibitor 3-Methyladenine (3-MA) (cat. No. M9281; Sigma-Aldrich; *n* = 24) or phosphate-buffered saline as vehicle (*n* = 24) 24 h later. The infusions (12 μl/d over a period of 7 days) were made using a 1007D Alzet osmotic minipump (Durect, Cupertino, CA, USA) at the following stereotaxic coordinates: 0.8 mm posterior to the bregma, 1.4 mm lateral to the bregma on the right side and 3.6 mm below the dura. The *in vivo* bioactivity of reagents in the present regimen has been verified by our previous study (Liao et al., 2013).

### Neurological evaluation

Neurological function was evaluated blindly in each group before and 1, 8, and 14 days after MCAO with a modified neurologic severity score (mNSS), which included a combination of motor and sensory functions, balance, and reflex tests (Chen et al., 2014). The mNSS was recorded from 0 (normal) to 18 (maximal deficit), with 13–18 as severe injury, 7–12 as moderate injury, and 1–6 as mild injury.

### Tissue preparation

Eight and 14 days after real or sham MCAO, 12 rats from each group were anesthetized and sacrificed. For H&E staining and immunofluorescence, the rat brains from six rats were transcardially perfused and post-fixed with 4% paraformaldehyde at 4°C for 8 h. Series of adjacent 10 μm coronal frozen sections were collected at the ipsilateral thalamus level. For western blot, the rat brains from the other six rats were transcardially perfused with heparinized saline at 4°C. The ipsilateral thalamus was quickly obtained and frozen in liquid nitrogen, and then stored at −80°C.

To observe the microstructure of tight junctions, small blocks from the ipsilateral thalamus (*n* = 3) were fixed, dehydrated, and embedded for transmission electron microscopy. Series of adjacent 80 nm sections were made using an Ultracut-E ultramicrotome (Reichert-Jung, Vienna, Austria), and viewed under a LM-10 electron microscope (Philips, Amsterdam, Holland) at 1,700 × magnification. Tight junctions appear as a series of discrete sites of apparent membrane fusion (kissing points) between the outer leaflets of the plasma membranes of adjacent cells.

### Cell culture and oxygen-glucose deprivation (OGD)

Rat brain microvascular endothelial cells (RBMVECs) (cat. No. R840-05a; Cell Application) were grown and maintained in Dulbecco's modified eagle medium (cat. No. 11885-084; Thermo Fisher Scientific) supplemented with 10% fetal bovine serum and 1% penicillin/streptomycin in a humidified incubator under 5% CO_2_ at 37°C. Cells were split at 70–80% confluence before the following experiments.

OGD is used to mimic ischemic conditions *in vitro* as previously described (Park et al., 2005). In brief, RBMVECs were gently washed twice with glucose-free Dulbecco's modified eagle medium (cat. No. 11966-025; Thermo Fisher Scientific), and placed in a modular chamber with dual flow meter (Billups-Rothenberg, Del Mar, CA, USA). Cells in the chamber were flushed with 95%N_2_/5% CO_2_ gas mixture at a flow rate of 4L/min for 10 min to create hypoxic conditions, and then incubated at 37°C for 1 h. Hypoxic conditions within the chamber were monitored using a gas analyzer (Coy Laboratory, Grass Lake, MI, USA). The extent of OGD-induced death of cells was dependent on the duration of OGD, and OGD for 1 h is at a critical threshold to induce pivotal signaling events for cells in the current method. Control cells were treated without OGD condition.

To elucidate the role of netrin-1 on RBMVECs and possible involvement of PI3K pathway, cells were pre-treated with 50 ng/mL netrin-1 (R&D System), 20 μmol/L PI3K inhibitor LY294002 (cat. No. L9908; Sigma-Aldrich), netrin-1 plus LY294002, or only equivalent amount of diluent solution for 2 h before OGD. The used concentrations of reagents were based on previous studies and were effective for its physiological function (Park et al., 2004; Wilson et al., 2006).

### UNC5H2 small interfering RNA (siRNA) transfection

UNC5H2 and scramble siRNA were designed by RiboBio Corporation (RiboBio, Guangzhou, China). Sequences corresponding to the siRNA of rat UNC5H2 were: sense, 5′GGAGGUACCCUUGGAUCAUdTdT3′; antisense, 5′AUGAUCCAAGGGUACCUCCdTdT3′. UNC5H2 siRNA-lipid or negative control siRNA-lipid complexes were made by adding siRNA to Lipofectamine RNAiMAX Reagent (cat. No. 13778; Thermo Fisher Scientific) diluted in Opti-MEM I reduced Serum medium (cat. No. 31985070; Thermo Fisher Scientific) to achieve a final concentration of 100 nmol/L. RBMVECs were incubated with 250 μL of siRNA-lipid complexes in a 6-well plate for 1 d at 37°C. The efficiency of transfection was validated by comparing the levels of UNC5H2 mRNA and protein between transfected and controlled cells by real-time PCR and western blot.

### Cell viability assay

Viable cells were detected by using a cell counting kit-8 (CCK-8, cat. No. CK04; Dojindo) and an Annexin V-FITC Kit (cat. No. AD10; Dojindo) following the instructions of manufacturer. For CCK-8 detection, 100 μl cell suspension (10^4^ cells/well) was dispensed in a 96-well plate and pre-incubated for 24 h in a humidified incubator with 5% CO2 at 37°C. Cells in each well were mixed with 10 μl CCK-8 solution and incubated for 4 h at 37°C in dark. Cell viability was decided by measuring the absorbance at 450 nm (ref. 650 nm) under a multi-mode Synergy HT microplate reader (BioTek, Winooski, VT, USA). The deeper color the culture medium presented, the more living cells there were. Blank control was performed by adding equivalent amount of CCK-8 solution into non-cell well. For Annexin V-FITC detection, cells were gently digested by 0.25% trypsin without EDTA and washed twice with 0.01 mol/L PBS, and then 5 × 10^5^ cells were collected by centrifugation at 1,000 rpm for 5 min. Cells were resuspended in 500 μl of 1 × Binding Buffer, and incubated with 5 μl of Annexin V-FITC and 5 μl of propidium iodide (PI) at room temperature for 15 min in dark. The green fluorescence of Annexin V-FITC binding and the red fluorescence of PI staining were respectively detected by an EPICS XL-MCL flow cytometry (Bechman Coulter, Brea, CA, USA). Viable cells were defined as FITC (–) and PI (–) cells. Control cells were treated with either Annexin V-FITC or PI staining, or non-staining of both.

### Immunofluorescence

#### In vivo

A mouse anti-rat endothelial cell antigen-1 (RECA-1) antibody (1:300; cat. No. NB100-64647; Novus Biologicals) was used to mark ECs in the ipsilateral thalamus. Sections were first incubated with RECA-1 antibody at 4°C overnight before incubated with species-specific Alexa Fluor® 555-conjugated anti-mouse (cat. No. 4409; CST) for 1 h. Thereafter, sections were mounted and analyzed under a fluorescence microscope with a Kontron IBAS 2.5 image system. Double immunofluorescence was performed in order to evaluate the permeability of BBB. Sections were first incubated with a mouse anti-albumin antibody (1:300; cat. No. sc-271605; Santa Cruz Biotechnology), and then combined with a rabbit anti-occludin antibody (1:100; cat. No. 71-1500; Invitrogen). Species-specific Alexa Fluor® 488-conjugated anti-mouse secondary antibody (cat. No. 4408; CST) and Alexa Fluor® 555-conjugated anti-rabbit secondary antibody (cat. No. 4413; CST) were used as described above.

#### In vitro

The endothelial attribute of cells was identified by using a rabbit anti-von Willebrand factor (vWF) antibody (1:200; cat. No. sc-14014; Santa Cruz Biotechnology). To detect netrin-1 receptors, RBMVECs were first incubated with a rabbit anti-DCC antibody (1:500; cat. No. SAB4500625; Sigma-Aldrich) or UNC5H2 antibody (1:300; cat. No. ab104871; Abcam) at 4°C overnight before incubated with species-specific Alexa Fluor 488-conjugated goat anti-rabbit secondary antibody (cat. No. 4412S; CST) for 1 h. Thereafter, cells were counterstained with Hochest 33258 solution (cat. No. 94403; Sigma-Aldrich), and analyzed as described above.

### Western blot

Homogenized brain tissue or cultured cells were lysed using RIPA Buffer (cat. No. 89900; Thermo Fisher Scientific) plus a 10 μl /mL protease inhibitor cocktail (cat. No. 78410; Thermo Fisher Scientific) on ice for 5 min. The pellet was simultaneously sonicated for 30 s with 50% pulse to increase yields. Then, the lysate was centrifuged at 14,000 g for 15 min at 4°C to obtain the supernatant sample. A standard curve by plotting an average blank-corrected 562 nm measurement for each BSA standard vs. its concentration in μl /mL was made using a BCA protein assay kit (cat. No. 23227; Thermo Fisher Scientific). The protein concentration of each sample was quantitated using the standard curve. Equivalent amount of proteins was resolved by electrophoresis using a sodium dodecyl sulfate–polyacrylamide gel and transferred to polyvinylidene fluoride membranes. Blots were incubated with a rabbit anti-vWF antibody (1:200; Santa Cruz Biotechnology), VEGF antibody (1:200; cat. No. sc-152; Santa Cruz Biotechnology), beclin-1 antibody (1:1,000; cat. No. 3495; CST), microtubule-associated protein light chain 3 (LC3) antibody (1:1,000; cat. No. 3868; CST), p62 antibody (1:1000; cat. No. 5114; CST), ZO-1 antibody (1:100; cat. No. 61-7300; Invitrogen), occludin antibody (1:100; Invitrogen), DCC antibody (1:1,000; Sigma-Aldrich), or UNC5H2 antibody (1:800; Abcam) at 4°C overnight. Antibody binding to blots was visualized on X-ray films using a HRP-linked rabbit IgG detection kit (1:2,000; cat. No. 7074; CST). The image program of Quantity One was used to measure the density of bands on western blots in a blinded manner. Results were expressed as a percentage of GAPDH to generate relative protein levels.

### Statistical analyses

All data were analyzed with SPSS (Windows version 15.0; SPSS Inc., Chicago, IL, USA). All analysis was performed in a blinded manner without knowledge of the treatment assignment. Numerical data were expressed as the mean ± standard deviation while ordinal data as the median with 25 and 75 percentile. A general linear model with analysis of variance was used to detect any intergroup differences. A two-tailed *P*-value of 0.05 or less was taken to infer statistical significance.

## Results

### MCAO resulted in secondary impairment of BBB in the ipsilateral thalamus

**Figure 1:**
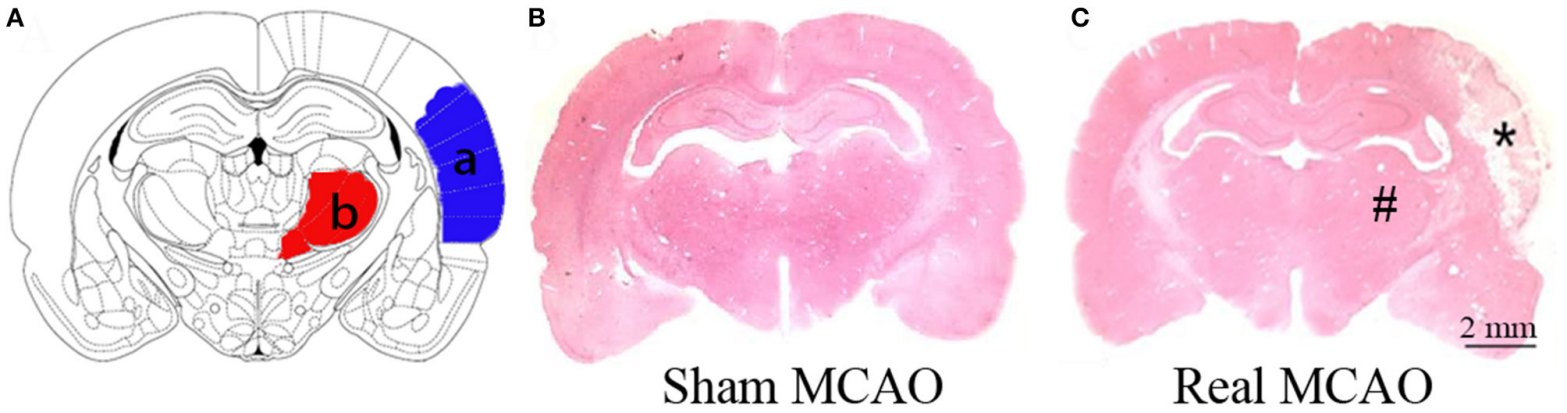
Cortical infarction after MCAO. **(A)** Schematic illustration of cortical infarction (a, blue) and the ipsilateral thalamus (b, red). **(B)** Normal brain tissue by H&E staining. **(C)** Focal infarction (^*^) was in the neocortex while the ipsilateral thalamus (#) was apparently unaffected 8 days after MCAO.

**Figure 2:**
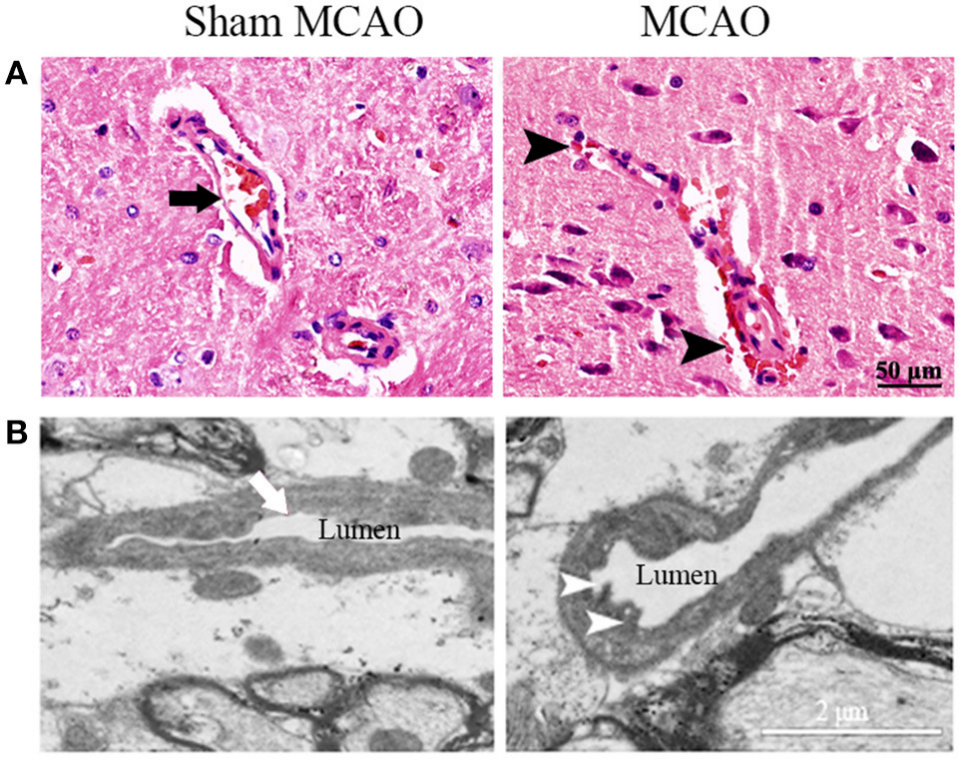
Representative images of microvascular structure in the ipsilateral thalamus. **(A)** Normal (arrow) and impaired microvascular structure (extravasation of erythrocyte, arrowheads) were showed by H&E staining. **(B)** Normal (arrow) and damaged tight junctions (discontinuous contact, arrowheads) were showed by transmission electron microscopy.

The infarction was consistently induced after MCAO in hypertensive rats, located in the ipsilateral primary and secondary somatosensory cortex sparing the thalamus, and caudate putamen (Figure 1). Nevertheless, extravasation of erythrocyte by H&E staining and damaged tight junctions by transmission electron microscopy were exhibited in the ipsilateral thalamus after MCAO, indicating the secondary impairment of BBB (Figure 2). There was no observed cerebral infarction in sham MCAO group.

### Netrin-1 improved neurological function after MCAO, inhibited by PI3K inhibitor 3-MA

**Figure 3:**
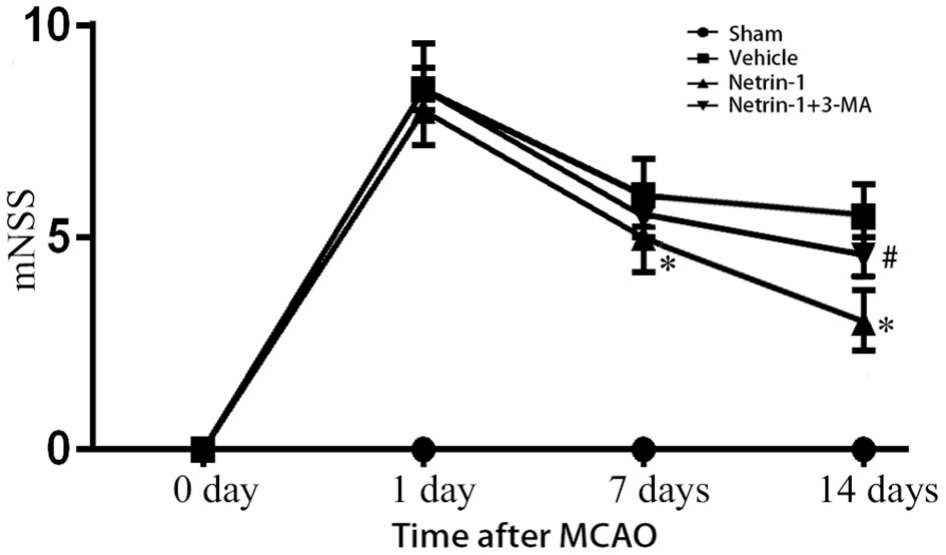
Neurological function assessed by mNSS. The scores were increased over different time points after MCAO, but significantly improved with netrin-1 infusion. The beneficial effect of netrin-1 was inhibited by 3-MA 14 days after MCAO. ^*^P < 0.05, vs. the vehicle group, # *P* < 0.05, vs. the netrin-1 group, *n* = 6.

Neurological function evaluated by mNSS was impaired after MCAO. Netrin-1 infusion improved the neurological impairment 8 and 14 days after MCAO compared with vehicle treatment. The difference was statistically significant. The beneficial effect of netrin-1 was inhibited by 3-MA 14 days after MCAO (Figure 3).

### Netrin-1 ameliorated secondary impairment of BBB and augmented cell survival, prohibited by PI3K inhibitors

**Figure 4:**
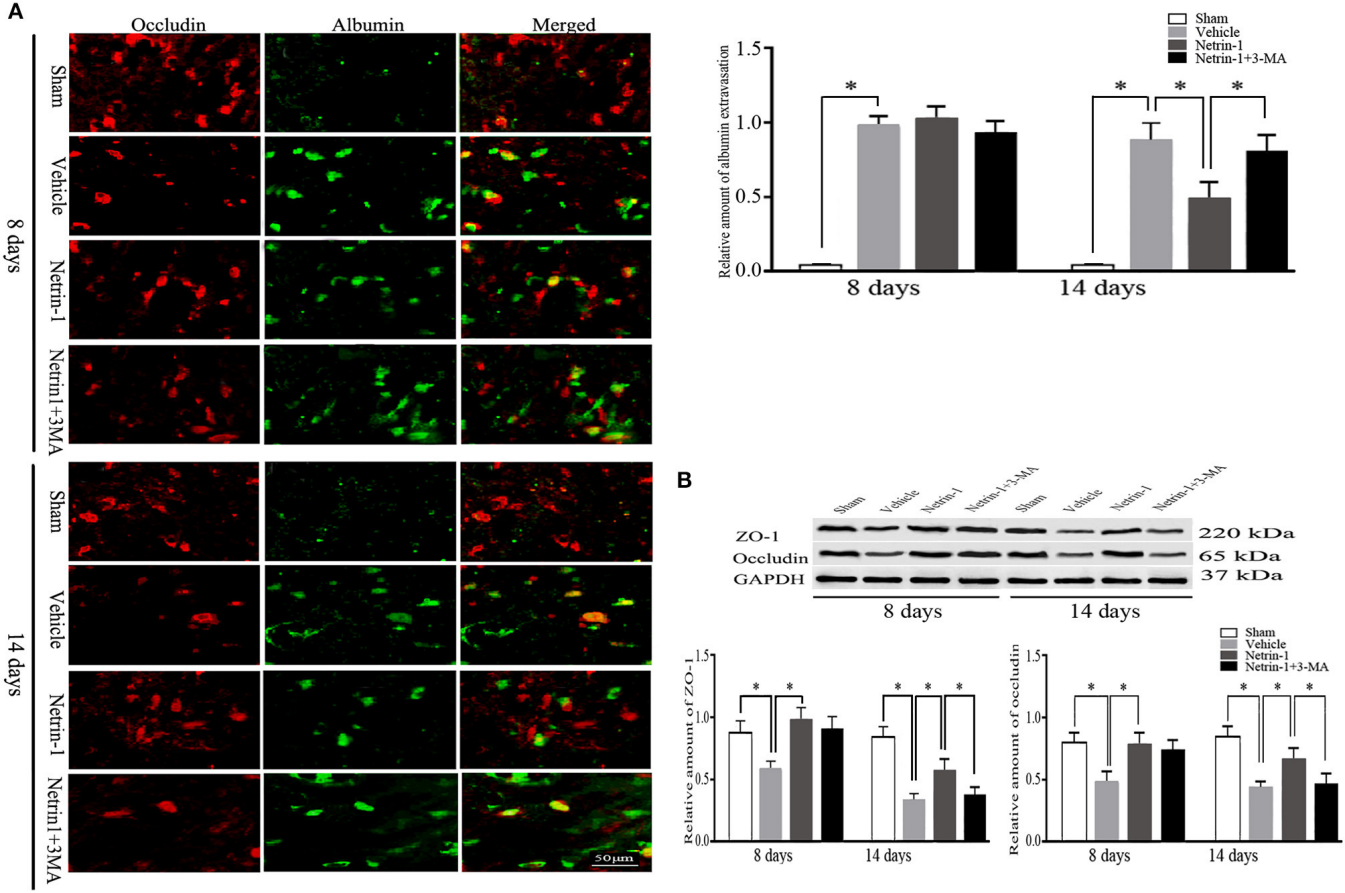
Secondary impairment of BBB in the ipsilateral thalamus. **(A)** Representative images of occludin (red) and extravasation of albumin (green), and relative amount of albumin extravasation by immunofluorescence. **(B)** Western blot analysis of tight junction protein ZO-1 and occludin. Extravasation of albumin and down-regulation of ZO-1 and occludin after MCAO were improved by netrin-1 infusion. PI3K inhibitor 3-MA prohibited the beneficial effect of netrin-1. ^*^P < 0.05, *n* = 6.

**Figure 5:**
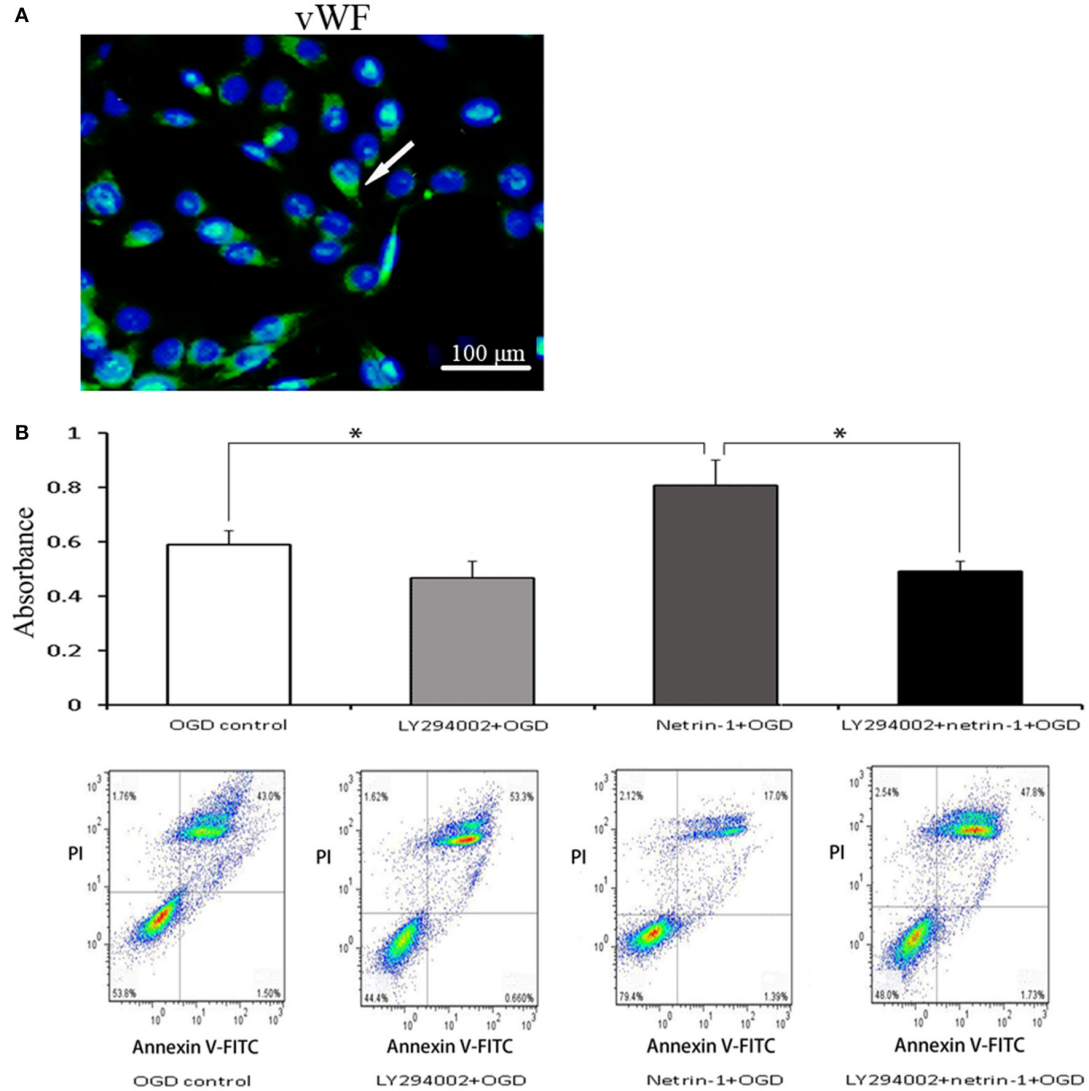
Characteristic and viability of RBMVECs. **(A)** RBMVECs were vWF-positive, indicating the attribute of ECs. Arrows indicates positive green signal by immunofluorescence. **(B)** Cell viability shown by CCK-8 and Annexin V-FITC kit. Netrin-1 (50 ng/mL) enhanced cell viability after OGD while PI3K inhibitor LY294002 prohibited the beneficial effect of netrin-1. ^*^P < 0.05.

Eight days after MCAO, the down-regulation of tight junction protein ZO-1 and occludin as well as extravasation of albumin was detected in the ipsilateral thalamus, both of which lasted until 14 days. Albumin showed a partial co-localization with occludin by double immunofluorescence. When compared with the vehicle group, netrin-1 infusion improved the permeability of BBB, represented by increased protein levels of ZO-1 and occludin 8 and 14 days after MCAO, and diminished extravasation of albumin at 14 days. Meanwhile, in vWF-stained RBMVECs, with the supplement of netrin-1 (50 ng/mL) before OGD, cell viability was significantly raised compared to the OGD control [0.81 ± 0.10 vs. 0.59 ± 0.05 by cell absorbance; 79.4 vs. 53.8% by the proportion of PI/Annexin V-FITC (–) cells]. By contrast, the beneficial effects of netrin-1 on BBB permeability and cell survival were prohibited by PI3K inhibitors 3-MA and LY294002 14 days after MCAO, shown by down-regulation of tight junction proteins and intensified extravasation of albumin, as wells as decreased cell viability (0.52 ± 0.03 and 48.0%, respectively), suggesting PI3K pathway was involved in the protective role of netrin-1 (Figures 4, 5).

**Figure 6:**
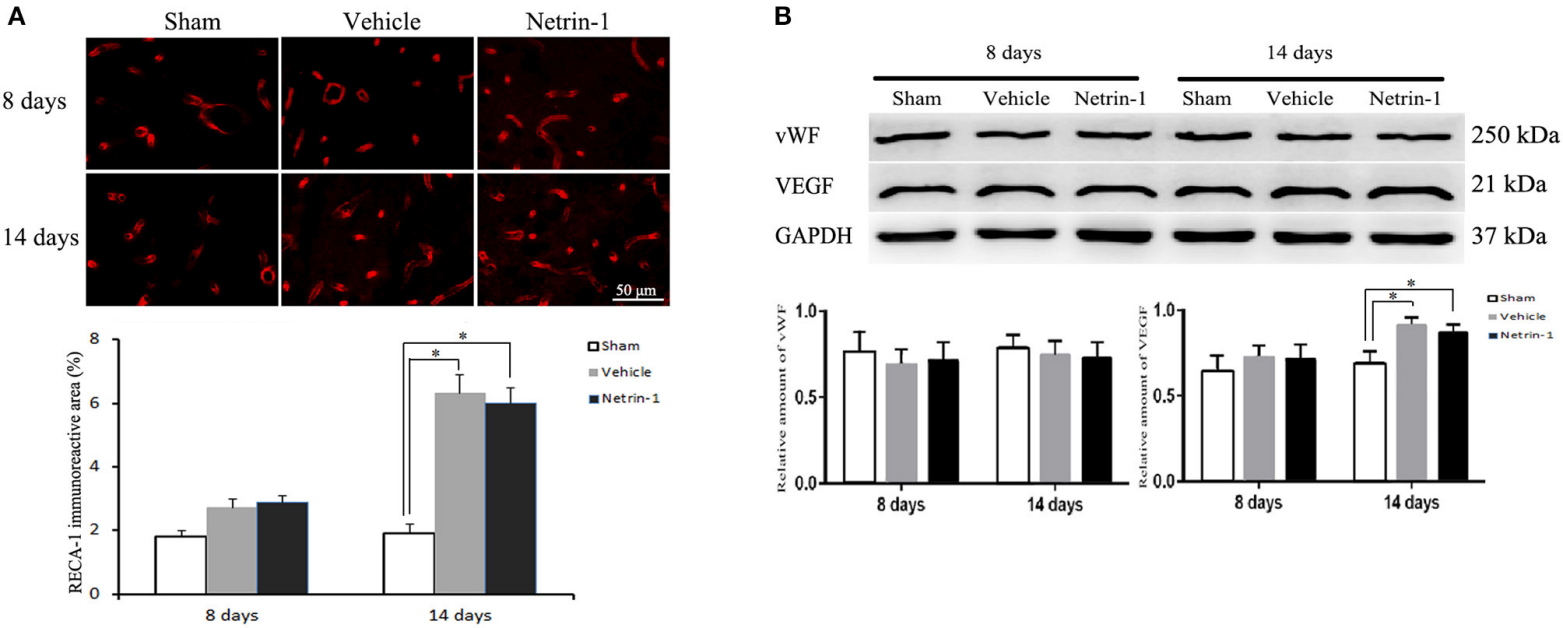
Detection of endothelial cells in the ipsilateral thalamus. **(A)** Representative images of RECA-1 (red) by immunofluorescence. **(B)** Western blot analysis. The positive area of RECA-1 and the protein level of VEGF were increased 14 days after MCAO. Netrin-1 infusion did not change the positive area of RECA-1 or the protein levels of vWF and VEGF after MCAO. ^*^P < 0.05, *n* = 6.

ECs in the ipsilateral thalamus were evaluated by detection of RECA-1, vWF, and VEGF. The positive area of RECA-1 and the protein level of VEGF were significantly increased 14 days after MCAO by immunofluorescence and western blot. Netrin-1 infusion did not make significant difference of RECA-1, vWF, or VEGF over different time points compared with the vehicle group (Figure 6).

### Netrin-1 enhanced autophagy activity, suppressed by PI3K inhibitors

**Figure 7:**
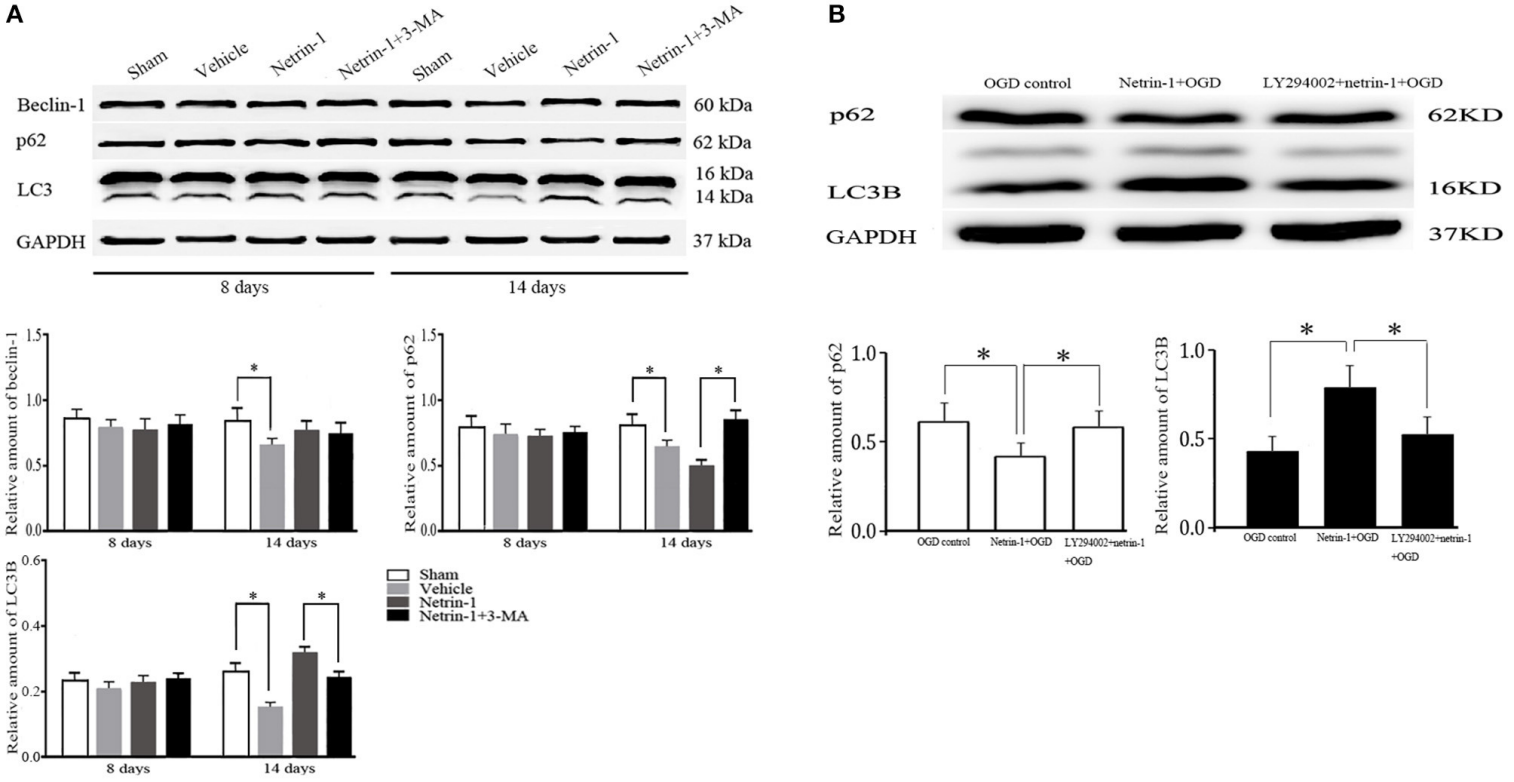
Western blot analysis of autophagy-related protein beclin-1, p62 and LC3. **(A)** Netrin-1 infusion increased autophagy activity 14 days after MCAO compared with the vehicle group, suppressed by PI3K inhibitor 3-MA. **(B)** Netrin-1 (50 ng/mL) enhanced autophagy activity after OGD while PI3K inhibitor LY294002 suppressed the effect of netrin-1. ^*^P < 0.05, *n* = 6.

The whole autophagy activity in the ipsilateral thalamus was reduced 14 days after MCAO by western blot, represented by reduced protein levels of LC3B, beclin-1, and p62. Netrin-1 infusion significantly increased the levels of LC3B and beclin-1, and decreased p62 14 days after MCAO compared with the vehicle group, indicating the activation of autophagy. Similarly, the supplement of netrin-1 (50 ng/mL) also significantly increased autophagy activity in OGD-treated cells. By contrast, the netrin-1-activated autophagy was suppressed by PI3K inhibitors 3-MA and LY294002, shown by diminished LC3B and augmented p62, implying that PI3K-mediated autophagy activity participated in the protective role of netrin-1 (Figure 7).

### The effects of netrin-1 on cell viability and autophagy were dependent on its UNC5H2 receptor

**Figure 8:**
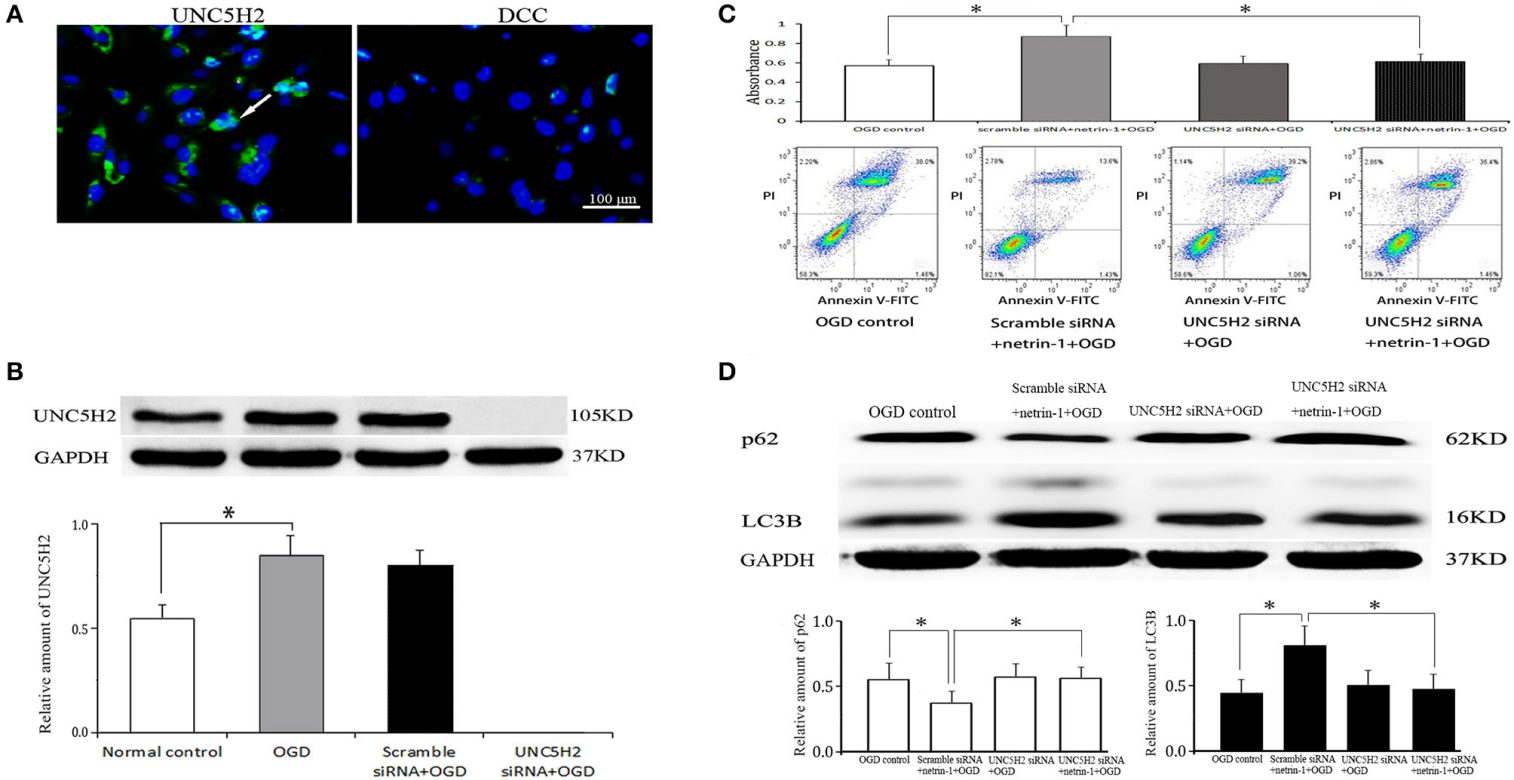
Netrin-1 receptors on RBMVECs and their function. **(A)** UNC5H2 receptor, not DCC receptor, was expressed on RBMVECs. Arrows indicates positive green signal by immunofluorescence. **(B)** Western blot analysis of UNC5H2 receptor. OGD-induced UNC5H2 was efficiently knocked down by UNC5H2 siRNA transfection. **(C)** Cell viability shown by CCK-8 and Annexin V-FITC kit. **(D)** Western blot analysis of autophagy-related protein p62 and LC3. UNC5H2 siRNA transfection did not change cell viability or autophagy activity after OGD, but suppressed the strengthened effects of netrin-1 (50 ng/mL) on cell viability and autophagy activity. ^*^P < 0.05.

UNC5H2 receptor, not DCC receptor, was distinctly expressed on RBMVECs. OGD for 1 h significantly up-regulated the protein level of UNC5H2 receptor compared with the normal control. UNC5H2 siRNA transfection efficiently knocked down the level of UNC5H2, but did not change cell viability or autophagy activity after OGD. In the meantime, after UNC5H2 siRNA transfection, cell viability and autophagy activity induced by netrin-1 were significantly suppressed, implying that the beneficial effects of netrin-1 required UNC5H2 receptor (Figure 8).

## Discussion

Secondary vascular and neuronal injury in the remote thalamus, which is not supplied by MCA, displays a time profile different from that in the primary infarction lesion (Zhang et al., 2012). In the present study with MCAO in renovascular hypertensive rats, the immunoreactivity of RECA-1 and the protein level of VEGF were increased in the ipsilateral thalamus 14 days after MCAO, inferring an enhancement of angiogenesis. Nevertheless, the protein level of vWF, a marker mainly expressed in mature ECs, remained unchanged. It can be deduced that the function of new-formed vessels was still imperfect. Indeed, we found that the function of BBB in the vessels was impaired after MCAO, represented by increased extravasation of albumin and damaged tight junctions. In the meantime, down-regulation of tight junction protein ZO-1 and occludin was also observed. Thus, secondary impairment of BBB in the ipsilateral thalamus could be disadvantage to neurological recovery post stroke.

Axon guidance cue netrin-1 has been shown as a survival factor for ECs through receptor-dependent pathways. In adult brain, focal newly-formed blood vessels facilitated by netrin-1 overexpression contained an intact EC monolayer surrounded by multiple cell layers (Fan et al., 2008). Netrin-1 also increased peri-infarct vessel density after ischemic insult and maintained vascular integrity against inflammatory injury (Sun et al., 2011; Podjaski et al., 2015). Nevertheless, in some cases, netrin-1 was found to block angiogenesis through its receptor of UNC5B (Lu et al., 2004; Larrivee et al., 2007). The dual function of netrin-1 has been attributed to its concentrations used and the specific type of receptors involved in different experimental conditions (Yang et al., 2007; Castets and Mehlen, 2010).

With exogenous netrin-1 protein continuously infused to the ventricle for 7 days after MCAO, we found the expression of ECs in the ipsilateral thalamus remained unchanged compared to the vehicle control, but neurological function was improved. Simultaneously, extravasation of albumin was decreased while the protein levels of ZO-1 and occludin were increased, implying that netrin-1 improved neurological function through the preservation of BBB but not angiogenesis after MCAO. We further performed *in vitro* culture of RBMVECs, and found that OGD-inhibited cell viability was enhanced after being treated with netrin-1 (50 ng/mL). Meanwhile, the expression of netrin-1 receptor UNC5H2, rather than DCC, was obviously elevated after OGD. After UNC5H2 siRNA transfection, the protective effect of netrin-1 on cell viability was significantly decreased compared with the control. Taken together, the data showed that the beneficial role of netrin-1 on BBB was achieved by up-regulating the levels of tight junction proteins and supporting endothelial survival, possibly through UNC5H2 receptor.

The underlying mechanism of netrin-1 on BBB maintenance is not clear. Studies in *Caenorhabditis elegans* found that autophagy-related kinase UNC-51 and its binding partner UNC-14 regulated the subcellular localization of netrin receptor UNC-5, and guided axon growth in *C. elegans* (Ogura and Goshima, 2006; Ogura et al., 2010). UNC5H2 also could trigger cell death through the activation of the serine-threonine protein kinase DAPk, which is also a critical regulator of autophagy (Guenebeaud et al., 2010; Levin-Salomon et al., 2014). These studies inferred a potential connection between autophagy and netrin-1/UNC5.

Autophagy is a key cellular process that preserves endothelial function (Mizushima and Komatsu, 2011; Rubinsztein et al., 2012). Impaired autophagy could affect the functionality of the vessel wall and initialize many vascular diseases. On the other hand, autophagy activation was proposed as a protective mechanism to recycle nutrients and to generate energy for maintenance of cell viability in unfavorable conditions (Meng et al., 2010; Zhang et al., 2010; Xie et al., 2011; Chen et al., 2013). In the present study, netrin-1 infusion enhanced autophagy activity in the ipsilateral thalamus 14 days after MCAO, in accordance with the improvement of BBB permeability. Furthermore, PI3K inhibitor 3-MA inhibited not only autophagy activity, but also the protective role of netrin-1 on BBB in the ipsilateral thalamus after MCAO. *In vitro*, netrin-1 enhanced autophagy activity and cell viability compared with the OGD-control. Nevertheless, the activated autophagy and cell viability were diminished when simultaneously mixed with the other PI3K inhibitor LY294002. After UNC5H2 siRNA transfection, both netrin-1-activated autophagy and cell survival were inhibited. Altogether, the data inferred that PI3K-mediated autophagy activity depending on UNC5H2 receptor could be involved in both the improvement of BBB function and the survival of ECs.

There were several limitations in our study. First, the ideal *in vitro* model of secondary injury has not been well established yet due to the unclarified mechanisms in remote loci. Nevertheless, the OGD-treated cell model *in vitro* used in this study still offered a method to study the underlying mechanism of secondary injury. Secondly, the level of UNC5H2 rather than DCC was clearly elevated in this study, the data, nonetheless, did not exclude the possibility that the beneficial effects of netrin-1 were mediated through other known netrin-1 receptors like neogenin and CD146, which were also expressed in ECs (Tsuchiya et al., 2007; Tu et al., 2015). Thirdly, overactivation of PI3K-mediated autophagy may undoubtedly cause cell death. It is not clear that in what range of netrin-1-activated autophagy is beneficial to cell survival. These issues remain to be elucidated in the future.

## Conclusion

In summary, the present study favors the results that netrin-1 ameliorated the impairment of BBB secondary to ischemic stroke by promoting tight junction function and endothelial survival. PI3K-mediated autophagy activation depending on UNC5H2 receptor could be an underlying mechanism. Future studies are warranted to explore the detailed pathway of netrin-1/UNC5H2 in autophagy process.

## Author contributions

JY and SL designed the work, wrote the manuscript, and interpreted the data; JY and CL performed the experiments; QD, JQ, KL, and HW analyzed the data.

### Conflict of interest statement

The authors declare that the research was conducted in the absence of any commercial or financial relationships that could be construed as a potential conflict of interest.

## Abbreviations

BBB: blood-brain barrier
DCC: deleted in colorectal cancer
UNC5H: uncoordinated gene 5H
ECs: endothelial cells
PI3K: phosphatidylinositol 3 kinase
MCAO: middle cerebral artery occlusion
3-MA: 3-Methyladenine
mNSS: modified neurologic severity score
OGD: oxygen-glucose deprivation
RBMVECs: rat brain microvascular endothelial cells
siRNA: small interfering RNA
PI: propidium iodide
RECA-1: rat endothelial cell antigen-1
vWF: von Willebrand factor
VEGF: vascular endothelial growth factor
LC3: microtubule-associated protein light chain 3.

## References

[B1] Abraham C. S. Harada N. Deli M. A. Niwa M. (2002). Transient forebrain ischemia increases the blood-brain barrier permeability for albumin in stroke-prone spontaneously hypertensive rats. Cell. Mol. Neurobiol. 22, 455–462. 10.1023/A:1021067822435 12507394 PMC11533779

[B2] Castets M. Mehlen P. (2010). Netrin-1 role in angiogenesis: to be or not to be a pro-angiogenic factor? Cell Cycle 9, 1466–1471. 10.4161/cc.9.8.11197 20372055

[B3] Chen G. Zhang W. Li Y. P. Ren J. G. Xu N. Liu H.. (2013). Hypoxia-induced autophagy in endothelial cells: a double-edged sword in the progression of infantile haemangioma? Cardiovasc. Res. 98, 437–448. 10.1093/cvr/cvt035 23408345

[B4] Chen X. R. Liao S. J. Ye L. X. Gong Q. Ding Q. Zeng J. S.. (2014). Neuroprotective effect of chondroitinase ABC on primary and secondary brain injury after stroke in hypertensive rats. Brain Res. 1543, 324–333. 10.1016/j.brainres.2013.12.002 24326094

[B5] Duering M. Righart R. Wollenweber F. A. Zietemann V. Gesierich B. Dichgans M. (2015). Acute infarcts cause focal thinning in remote cortex via degeneration of connecting fiber tracts. Neurology 84, 1685–1692. 10.1212/WNL.0000000000001502 25809303 PMC4409580

[B6] Fan Y. Shen F. Chen Y. Hao Q. Liu W. Su H.. (2008). Overexpression of netrin-1 induces neovascularization in the adult mouse brain. J. Cereb. Blood Flow Metab. 28, 1543–1551. 10.1038/jcbfm.2008.39 18461079 PMC2581494

[B7] Guenebeaud C. Goldschneider D. Castets M. Guix C. Chazot G. Delloye-Bourgeois C.. (2010). The dependence receptor UNC5H2/B triggers apoptosis via PP2A-mediated dephosphorylation of DAP kinase. Mol. Cell 40, 863–876. 10.1016/j.molcel.2010.11.021 21172653

[B8] Krueger M. Bechmann I. Immig K. Reichenbach A. Hartig W. Michalski D. (2015). Blood-brain barrier breakdown involves four distinct stages of vascular damage in various models of experimental focal cerebral ischemia. J. Cereb. Blood Flow Metab. 35, 292–303. 10.1038/jcbfm.2014.199 25425076 PMC4426746

[B9] Larrivee B. Freitas C. Trombe M. Lv X. Delafarge B. Yuan L.. (2007). Activation of the UNC5B receptor by Netrin-1 inhibits sprouting angiogenesis. Genes Dev. 21, 2433–2447. 10.1101/gad.437807 17908930 PMC1993874

[B10] Levin-Salomon V. Bialik S. Kimchi A. (2014). DAP-kinase and autophagy. Apoptosis 19, 346–356. 10.1007/s10495-013-0918-3 24264886

[B11] Li J. J. Xing S. H. Zhang J. Hong H. Li Y. L. Dang C.. (2011). Decrease of tight junction integrity in the ipsilateral thalamus during the acute stage after focal infarction and ablation of the cerebral cortex in rats. Clin. Exp. Pharmacol. Physiol. 38, 776–782. 10.1111/j.1440-1681.2011.05591.x 21851377

[B12] Liao S. J. Gong Q. Chen X. R. Ye L. X. Ding Q. Zeng J. S.. (2013). Netrin-1 rescues neuron loss by attenuating secondary apoptosis in ipsilateral thalamic nucleus following focal cerebral infarction in hypertensive rats. Neuroscience 231, 225–232. 10.1016/j.neuroscience.2012.11.059 23232257

[B13] Liman T. G. Endres M. (2012). New vessels after stroke: postischemic neovascularization and regeneration. Cerebrovasc. Dis. 33, 492–499. 10.1159/000337155 22517438

[B14] Ling L. Zeng J. Pei Z. Cheung R. T. Hou Q. Xing S.. (2009). Neurogenesis and angiogenesis within the ipsilateral thalamus with secondary damage after focal cortical infarction in hypertensive rats. J. Cereb. Blood Flow Metab. 29, 1538–1546. 10.1038/jcbfm.2009.76 19536072

[B15] Lu X. Le Noble F. Yuan L. Jiang Q. De Lafarge B. Sugiyama D.. (2004). The netrin receptor UNC5B mediates guidance events controlling morphogenesis of the vascular system. Nature 432, 179–186. 10.1038/nature03080 15510105

[B16] Lv J. Sun X. Ma J. Ma X. Zhang Y. Li F.. (2015). Netrin-1 induces the migration of Schwann cells via p38 MAPK and PI3K-Akt signaling pathway mediated by the UNC5B receptor. Biochem. Biophys. Res. Commun. 464, 263–268. 10.1016/j.bbrc.2015.06.140 26116534

[B17] Meng N. Wu L. Gao J. Zhao J. Su L. Su H.. (2010). Lipopolysaccharide induces autophagy through BIRC2 in human umbilical vein endothelial cells. J. Cell. Physiol. 225, 174–179. 10.1002/jcp.22210 20458734

[B18] Mizushima N. Komatsu M. (2011). Autophagy: renovation of cells and tissues. Cell 147, 728–741. 10.1016/j.cell.2011.10.026 22078875

[B19] Moore S. W. Tessier-Lavigne M. Kennedy T. E. (2007). Netrins and their receptors. Adv. Exp. Med. Biol. 621, 17–31. 10.1007/978-0-387-76715-4_2 18269208

[B20] Navankasattusas S. Whitehead K. J. Suli A. Sorensen L. K. Lim A. H. Zhao J.. (2008). The netrin receptor UNC5B promotes angiogenesis in specific vascular beds. Development 135, 659–667. 10.1242/dev.013623 18223200 PMC2612632

[B21] Nguyen A. Cai H. (2006). Netrin-1 induces angiogenesis via a DCC-dependent ERK1/2-eNOS feed-forward mechanism. Proc. Natl. Acad. Sci. U.S.A. 103, 6530–6535. 10.1073/pnas.0511011103 16611730 PMC1458918

[B22] Ogura K. Goshima Y. (2006). The autophagy-related kinase UNC-51 and its binding partner UNC-14 regulate the subcellular localization of the Netrin receptor UNC-5 in *Caenorhabditis elegans*. Development 133, 3441–3450. 10.1242/dev.02503 16887826

[B23] Ogura K. Okada T. Mitani S. Gengyo-Ando K. Baillie D. L. Kohara Y.. (2010). Protein phosphatase 2A cooperates with the autophagy-related kinase UNC-51 to regulate axon guidance in *Caenorhabditis elegans*. Development 137, 1657–1667. 10.1242/dev.050708 20392746 PMC3188576

[B24] Park E. M. Cho B. P. Volpe B. T. Cruz M. O. Joh T. H. Cho S. (2005). Ibuprofen protects ischemia-induced neuronal injury via up-regulating interleukin-1 receptor antagonist expression. Neuroscience 132, 625–631. 10.1016/j.neuroscience.2005.01.021 15837124

[B25] Park K. W. Crouse D. Lee M. Karnik S. K. Sorensen L. K. Murphy K. J.. (2004). The axonal attractant Netrin-1 is an angiogenic factor. Proc. Natl. Acad. Sci. U.S.A. 101, 16210–16215. 10.1073/pnas.0405984101 15520390 PMC528958

[B26] Podjaski C. Alvarez J. I. Bourbonniere L. Larouche S. Terouz S. Bin J. M.. (2015). Netrin 1 regulates blood-brain barrier function and neuroinflammation. Brain 138, 1598–1612. 10.1093/brain/awv092 25903786 PMC4614143

[B27] Prakash R. Carmichael S. T. (2015). Blood-brain barrier breakdown and neovascularization processes after stroke and traumatic brain injury. Curr. Opin. Neurol. 28, 556–564. 10.1097/WCO.0000000000000248 26402408 PMC5267616

[B28] Rajasekharan S. Kennedy T. E. (2009). The netrin protein family. Genome Biol. 10:239. 10.1186/gb-2009-10-9-239 19785719 PMC2768972

[B29] Rubinsztein D. C. Codogno P. Levine B. (2012). Autophagy modulation as a potential therapeutic target for diverse diseases. Nat. Rev. Drug Discov. 11, 709–730. 10.1038/nrd3802 22935804 PMC3518431

[B30] Seevinck P. R. Deddens L. H. Dijkhuizen R. M. (2010). Magnetic resonance imaging of brain angiogenesis after stroke. Angiogenesis 13, 101–111. 10.1007/s10456-010-9174-0 20552268 PMC2911530

[B31] Sun H. Le T. Chang T. T. Habib A. Wu S. Shen F.. (2011). AAV-mediated netrin-1 overexpression increases peri-infarct blood vessel density and improves motor function recovery after experimental stroke. Neurobiol. Dis. 44, 73–83. 10.1016/j.nbd.2011.06.006 21726647 PMC3179859

[B32] Tang X. Jang S. W. Okada M. Chan C. B. Feng Y. Liu Y.. (2008). Netrin-1 mediates neuronal survival through PIKE-L interaction with the dependence receptor UNC5B. Nat. Cell Biol. 10, 698–706. 10.1038/ncb1732 18469807 PMC2839190

[B33] Tsuchiya A. Hayashi T. Deguchi K. Sehara Y. Yamashita T. Zhang H.. (2007). Expression of netrin-1 and its receptors DCC and neogenin in rat brain after ischemia. Brain Res. 1159, 1–7. 10.1016/j.brainres.2006.12.096 17574219

[B34] Tu T. Zhang C. Yan H. Luo Y. Kong R. Wen P.. (2015). CD146 acts as a novel receptor for netrin-1 in promoting angiogenesis and vascular development. Cell Res. 25, 275–287. 10.1038/cr.2015.15 25656845 PMC4349246

[B35] Weinstein B. M. (2005). Vessels and nerves: marching to the same tune. Cell 120, 299–302. 10.1016/j.cell.2005.01.010 15707889

[B36] Wilson B. D. Ii M. Park K. W. Suli A. Sorensen L. K. Larrieu-Lahargue F.. (2006). Netrins promote developmental and therapeutic angiogenesis. Science 313, 640–644. 10.1126/science.1124704 16809490 PMC2577078

[B37] Xie Y. You S. J. Zhang Y. L. Han Q. Cao Y. J. Xu X. S.. (2011). Protective role of autophagy in AGE-induced early injury of human vascular endothelial cells. Mol. Med. Rep. 4, 459–464. 10.3892/mmr.2011.460 21468592

[B38] Xiong Y. Mahmood A. Chopp M. (2010). Angiogenesis, neurogenesis and brain recovery of function following injury. Curr. Opin. Investig. Drugs 11, 298–308. 20178043 PMC2836170

[B39] Yang Y. Zou L. Wang Y. Xu K. S. Zhang J. X. Zhang J. H. (2007). Axon guidance cue Netrin-1 has dual function in angiogenesis. Cancer Biol. Ther. 6, 743–748. 10.4161/cbt.6.5.3976 17387275

[B40] Zeng J. Zhang Y. Mo J. Su Z. Huang R. (1998). Two-kidney, two clip renovascular hypertensive rats can be used as stroke-prone rats. Stroke 29, 1708–1713. discussion: 1713–1704. 10.1161/01.STR.29.8.1708 9707215

[B41] Zhang J. Zhang Y. Xing S. Liang Z. Zeng J. (2012). Secondary neurodegeneration in remote regions after focal cerebral infarction: a new target for stroke management? Stroke 43, 1700–1705. 10.1161/STROKEAHA.111.632448 22492515

[B42] Zhang Y. L. Cao Y. J. Zhang X. Liu H. H. Tong T. Xiao G. D.. (2010). The autophagy-lysosome pathway: a novel mechanism involved in the processing of oxidized LDL in human vascular endothelial cells. Biochem. Biophys. Res. Commun. 394, 377–382. 10.1016/j.bbrc.2010.03.026 20223224

